# Assessment of post-laparotomy pain in laboratory mice by telemetric recording of heart rate and heart rate variability

**DOI:** 10.1186/1746-6148-3-16

**Published:** 2007-08-02

**Authors:** Margarete Arras, Andreas Rettich, Paolo Cinelli, Hans P Kasermann, Kurt Burki

**Affiliations:** 1 University of Zurich, Institute of Laboratory Animal Science, Sternwartstr. 6, CH – 8091 Zurich, Switzerland; 2 University of Zurich, Institute of Laboratory Animal Science, Winterthurerstr. 190, CH – 8057 Zurich, Switzerland

## Abstract

**Background:**

Pain of mild to moderate grade is difficult to detect in laboratory mice because mice are prey animals that attempt to elude predators or man by hiding signs of weakness, injury or pain. In this study, we investigated the use of telemetry to identify indicators of mild-to-moderate post-laparotomy pain.

**Results:**

Adult mice were subjected to laparotomy, either combined with pain treatment (carprofen or flunixin, 5 mg/kg s/c bid, for 1 day) or without pain relief. Controls received anesthesia and analgesics or vehicle only. Telemetrically measured locomotor activity was undisturbed in all animals, thus confirming that any pain experienced was of the intended mild level. No symptoms of pain were registered in any of the groups by scoring the animals' outer appearance or spontaneous and provoked behavior. In contrast, the group receiving no analgesic treatment after laparotomy demonstrated significant changes in telemetry electrocardiogram recordings: increased heart rate and decreased heart rate variability parameters pointed to sympathetic activation and pain lasting for 24 hours. In addition, core body temperature was elevated. Body weight and food intake were reduced for 3 and 2 days, respectively. Moreover, unstructured cage territory and destroyed nests appeared for 1–2 days in an increased number of animals in this group only. In controls these parameters were not affected.

**Conclusion:**

In conclusion, real-time telemetric recordings of heart rate and heart rate variability were indicative of mild-to-moderate post-laparotomy pain and could define its duration in our mouse model. This level of pain cannot easily be detected by direct observation.

## Background

Laboratory mice are currently the most widely used animal species in biomedical research. Their popularity is due to the availability of a wealth of spontaneous or experimentally induced mutants, allowing the *in vivo *functions of single genes to be studied. Therefore, mice provide powerful models with which to explore the regulation of cellular and physiologic processes. Mice are also increasingly used in complex investigations requiring additional surgical intervention. Doubtless, post-operative pain relief following surgical procedures such as laparotomy would seem to be necessary, but is often omitted as documented in a recent survey [1]. A reason given for withholding analgesics from laboratory rodents is that no signs of pain were observed and therefore analgesics were considered to be unnecessary.

Indeed, after minor surgery, signs of pain are barely observable in mice. From post-operative monitoring, which usually consists of visual inspection of the animal's appearance, posture, and spontaneous behavior, only the generalized, well-known symptoms of considerable or severe pain and suffering can be detected, e.g., piloerection, unkempt coat/rough hair, hunched posture, apathy, aggression, and self-mutilation [2-5].

An explanation for the phenomenon that pain of moderate grades is not visible in mice is that prey animals live in constant fear of falling victim to their enemies, therefore they tend to show as few signs of disease, suffering or weakness as possible. This instinctive strategy is intended to avoid attracting the attention of predators, including man. Therefore, during animal experiments, or even when a person is simply present in the room, the mouse will hide signs of pain [5-7], making monitoring of low-to-moderate pain difficult.

This problem can be circumvented by using telemetry, which allows monitoring without the presence of the investigator in the vicinity of the animal. Using commercially available radiotelemetric transmitters, physiological and behavioral parameters can be recorded in real-time over several days after surgery in rodents [8]. Such recordings provide information about the time course of disturbances in these parameters that cannot be obtained with such accuracy from retrospective measurements such as loss of body weight and reduction in food intake.

Telemetry in mice to measure physiological parameters such as heart rate (HR), core body temperature (BT), or blood pressure has been established. These latter parameters have been recommended as additional indices to appearance and behavior in determining pain scales for animals [3,6,7,9]. Since these measures are not specific to pain per se, their relevance in the assessment of pain must be worked out with specific types of pain excluding external influences. In this study, we aimed to identify telemetrically measurable indicators of mild-to-moderate post-laparotomy pain in mice. In addition, two regimens for pain relief were assessed.

## Results

### Animals and housing conditions

Twenty-six male HsdHan:NMRI mice were obtained from a commercial supplier (Harlan, Horst, The Netherlands). The mice were free of the viral, bacterial, and parasitic pathogens listed in the recommendations of the Federation of European Laboratory Animal Science Association [10]. Their health status was monitored by a sentinel program throughout the experiments. Animals were obtained at the age of 4 weeks. Telemetric transmitters were implanted after an adaptation period of 4 weeks, i.e., at 8 weeks of age. The experiments were conducted when the mice were aged 4–6 months, with body weights ranging from 40 to 54 g. In general, each male was housed together with an adult, ovarectomized female companion mouse, except that each animal was housed individually for two weeks after transmitter implantation and during the experiments. Housing in compatible pairs with an infertile female allowed normal social interaction of the experimental mature males while production of unwanted progeny was prevented. During the recovery phase from surgical interventions (transmitter implantation, vasectomy), the female companion was suspected to disturb healing processes and influence measurements (e.g. nest building). To exclude the impact of the companions' actions on recovery and read out, the experimental animals were housed individually during transient time periods.

Animals were kept in type 3 filter-top clear-transparent plastic cages (425 mm × 266 mm × 150 mm, floor area 820 cm^2^) with autoclaved dust-free sawdust bedding (80–90 g/cage) and autoclaved hay (18–20 g/cage) as nesting material. They were fed a pelleted and extruded mouse diet (Kliba No. 3431, Provimi Kliba, Kaiseraugst, Switzerland) ad libitum and had unrestricted access to sterilized drinking water. The light/dark cycle in the room consisted of 12/12 h (07:00–19:00) with artificial light (40 Lux in the cage). The temperature was 21 ± 1°C, with a relative humidity of 50 ± 5%, and with 15 complete changes of filtered air per hour (HEPA H 14 filter); the air pressure was controlled at 50 Pa. The animal room was insulated to prevent electronic noise. Disturbances (e.g., visitors or unrelated experimental procedures) were not allowed. To avoid interfering influences, all necessary husbandry and management procedures were completed in the room at least 3 days before starting an experiment.

Housing and experimental protocols were approved by the Cantonal Veterinary Department, Zurich, Switzerland, under license no. ZH 82/2004 and were in accordance with Swiss Animal Protection Law. Housing and experimental procedures were also in accordance with the *Guide for the Care and Use of Laboratory Animals *(Institute of Laboratory Animal Resources, National Research Council, National Academy of Sciences, 1996).

### Preliminary transmitter implantation

TA10ETA-F20 transmitters (Data Sciences International, St. Paul, MN, USA), which are able to process heart rate (HR), electrocardiogram (ECG), core body temperature (BT), and locomotor activity (ACT) in freely moving mice, were implanted as previously described [11]. The mice were anesthetized by inhalation of the volatile anesthetic sevoflurane (Sevorane™, Abbott, Cham, Switzerland) at a concentration of 5–8% in 100% oxygen at a flow rate of 200 ml/min. The anesthetic gas was administered with a nose mask. Ketamine (Ketasol-100™, Dr. Graub, Bern, Switzerland) was injected subcutaneously as pre-medication at a dosage of 45 mg/kg body weight as pre-emptive analgesia. The abdomen was opened by mid-line laparotomy and the telemetric transmitter body was implanted under aseptic conditions into the abdominal cavity of the mouse. One telemetry lead was tunneled subcutaneously from the thorax to the neck, where the wired loop electrode was fixed between the muscles located to the right of the trachea. The other wired loop lead was sutured to the xiphoid process with a silk thread. The muscle layers and the skin were then closed with resorbable sutures. Post-operative pain was treated with buprenorphine (Temgesic™, Reckitt and Colman Products Ltd., Hull, England), at a dose of 0.1 mg/kg body weight, injected subcutaneously twice per day for 4 days. Animals were monitored daily for 10 days after the surgery for appearance, posture, spontaneous moving behavior, body weight, and food and water consumption. After transmitter implantation, the mice had a period of 8 weeks convalescence until the start of the first experiment.

### Experimental design

Six experiments were performed: unilateral laparotomy either without subsequent analgesic treatment or with carprofen or flunixin treatment. Surgery was omitted in control groups, which received only anesthesia and injections of vehicle, carprofen or flunixin.

Mice were distributed randomly with regard to age and experiment. Each experiment was performed on 2–3 dates to reach a group size of 8 animals. For mice that underwent more than one experiment (26 mice/48 experiments), a break of at least two weeks between experiments was provided.

To avoid any influence of the circadian rhythm, the anesthesia, laparotomy and weighing procedures were conducted between 17:30 and 18:30 h, i.e., at the end of the light phase. All interventions were carried out in the animal room to preclude disturbance by transport and to ensure constant conditions.

### Method of establishing baseline values

To start an experiment, the transmitter-bearing male was separated from its infertile female companion and put in a fresh cage. For three days, the animal was allowed to settle in to the new territory and stabilize in single housing conditions without being disturbed.

Measurements were then started by obtaining the individual's baseline values for three consecutive days before laparotomy.

### Anesthesia and laparotomy experiments

For laparotomy and control experiments, animals were anesthetized for 10–11 minutes with sevoflurane (Sevorane™, Abbott, Cham, Switzerland) via a nose cone as follows: the animal was taken out of its cage, placed on the cage lid, restrained by the scruff of the neck and transferred onto an autoclaved, heated (39°C) operating table. There, its nose was placed in a face mask in which sevoflurane (7%) in oxygen (100%) was delivered (flow rate 200 ml/min) by an inhalation anesthesia device equipped with a vaporizer and a purpose-made gas delivery and scavenging system. After 10–15 seconds, the mouse lost the righting reflex and was turned onto its back, while its nose remained in the cone. Eyes were protected with ointment (Vitamine A, Bausch & Lomb, Steinhausen, Switzerland) and the lower abdominal region of the body was disinfected with alcohol (80%). After 5 minutes of anesthesia, the analgesic or vehicle injection was administered subcutaneously in the lateral abdominal region. The vehicle consisted of 2 μl phosphate buffered saline (PBS)/g body weight. Carprofen (Rimadyl™, Pfizer Inc., NY, USA) and flunixin (Biokema Flunixine™, Biokema SA, Crissier-Lausanne, Switzerland) were diluted in PBS to be applied at a dosage of 5 mg/kg body weight at the adequate volume of 2 μl/g body weight. Anesthesia was completed in the control groups without further intervention.

For laparotomy, the left or right inguinal region of the abdomen was opened with a small cut (<0.5 cm) in the skin and the muscular abdominal wall using sterile surgical instruments. The spermatic duct was ligated twice with a silk thread (6-0, Perma-Hand™ Seide, Ethicon, Norderstedt, Germany) and cut between the sutures, thus conducting unilateral vasectomy. The muscle layers were closed with resorbable sutures (6-0, Vicryl™, Ethicon, Norderstedt, Germany) and the skin restored with staples (Precise™, 3 M Health Care, St. Paul, MN, USA). Surgery was completed within 6–7 minutes.

When anesthesia was finished, the mouse was put back in its home cage, where it was able to move and orientate within the following 5–10 minutes.

All manipulations were completed by 18:30 h and the investigator had left the animal room before the night phase began. Twelve hours after the first injection of vehicle or analgesics, a second subcutaneous injection of the same substance and dosage was administered.

Data were collected for three days after laparotomy and anesthesia.

### Methods of data acquisition

Measurements started with the first baseline recordings at 8 weeks after transmitter implantation. Data were collected over 3 days for baseline values and after an experiment.

Telemetric measurements were processed using the Dataquest LabPRO program, version 3.11. The telemetric transmitter was switched on by touching the animal with a magnet; signals were detected by a receiver plate placed underneath the animal's cage.

HR values and ECG curves were recorded for 30 seconds every 5 minutes (sampling frequency 1000 Hz). ECG curves were subjected to a time domain analysis of heart rate variability (HRV) as defined [12,13]. The interbeat interval (IBI) and the standard deviation of interbeat interval (SDNN) were calculated from each segment measured. ACT was recorded continuously and stored at 5 minute intervals. BT was sampled for 10 seconds every 5 minutes.

Values of body weight, food and water consumption were established by weighing the animal, the food pellets and water bottle daily using a precision balance (PR 2003 Delta Range, Mettler-Toledo AG, Greifensee, Switzerland) especially designed to weigh moving animals.

The weighing procedure normally provokes the mouse to express reactions such as flight or attention, which are particularly apparent after the animal is returned to its cage. Therefore, mice were observed for 20–30 seconds before, during and after weighing to register spontaneous and provoked behavior and movements. These behaviors were scored together with clinical findings on the mouse's body once per day and additionally at 12 hours after laparotomy and anesthesia in order to identify signs of pain or disease. The parameters used for this observation of the animal are defined in the score sheet (Table 1). Each individual animal was scored 0 or 1.

**Table 1:** Scoring for examination of the animal and its cage

	Score 0	Score 1
**Observation of the animal**		
Spontaneous behavior	Sleeping, resting, digging, running, walking, rearing, climbing, eating, drinking, grooming, sniffing	Sudden movements, backwards movements, transient involuntary muscular contraction of any body part, kicking with hind paws, licking/biting the wound
Posture	Lying, sitting, moving,	Hunched, arched back, crouched
Breathing	Undisturbed, regular	Exerted, irregular
Coat condition	Clean, smooth, well-groomed	Ruffled, dirty, unkempt, piloerection, hair loss (alopecia)
Eyes	Clear, bright	Discharge
Body condition	Good, unchanged as judged from external appearance	Sunken flanks, swollen areas, ascites
Wound	Clean, dry, smooth	Dirty, bloody, uncleaned, signs of self-injury, signs of inflammation or necrosis, i.e., unusual color (e.g., red, pale) or swollen
Behavior after provocation/weighing	Alert, ready to take flight	Apathetic, sedated, highly aggressive, increased vocalization
Movement after provocation/weighing	No aberration in moving pattern	Decelerated/slowed, crawling, immobile, lameness, tiptoe gait

**Appearance of cage**		
Condition of nest	A nest clearly identifiable	Either no nest identifiable or multiple fragmentary nest-like resting places at different locations
Condition of territory	Cage area clearly structured, i.e., obvious areas for defecation and sleeping	Areas of defecation and sleeping indistinguishable, feces either adhering to nesting materials or not visible

The appearance of the cage, with a detailed description of its overall territorial structure and the condition of the nest was sketched on paper. This was carried out at 12-hour intervals without opening the cage or disturbing the mouse.

### Methods of data analysis

Baseline telemetric recordings were recorded for 3 days to assess whether the individual's data were stable from day to day. The means of the dark and light phase from the final day of baseline recording was used as each individual's baseline data. For each day after laparotomy/anesthesia, the mean of the dark and light phase was compared with the individual's baseline data, resulting in delta values. For HR, 3-hour means were additionally calculated by the same method (comparison with the corresponding baseline daytime data for each individual) from the first day after an experiment to allow construction of more detailed time courses of changes.

Baseline data for body weight, food and water consumption were summarized as the average of three days and compared with each day after laparotomy/anesthesia for each individual. Results are presented as the percentage deviation (delta).

Telemetric and weighing results are presented as mean ± standard error of mean (SEM).

Statistical analysis of telemetric data, as well as daily body weights and food and water consumption, was carried out using the program SPSS for Windows, version 13.0. The values obtained after anesthesia/laparotomy were compared with the individual's baseline values using two-tailed paired Student's *t*-test, with Bonferroni correction for multiple comparisons. Since three comparisons were made for 3-hour means of HR and for body weight, and food and water consumption, the alpha level was set at 0.05/3 = 0.0166 resulting in significance if the value of *P *was ≤ 0.016. Six comparisons were performed for the means of the dark and light phase, therefore the alpha level was set at 0.05/6 = 0.0083, with significance if the value of *P *was ≤ 0.008.

The sketches of the appearance of the cage were analyzed by a blinded investigator. The overall structure of the cage was assessed by the visibility of fixed areas for sleeping and/or defecation vs. lack of such structuring. The condition of the nest was assessed with focus on whether a nest was clearly identifiable or not. The sketches were scored as described in Table 1 and scores were finally condensed as the number of animals arranging their territory and nest in a specific manner (Table 2).

**Table 2:** Number of cages out of 8 estimated as Score 1 at the time points indicated.

	Laparotomy without pain treatment		Laparotomy with carprofen		Laparotomy with flunixin		control: anesthesia with vehicle		control: anesthesia with carprofen		control: anesthesia with flunixin	
	
	nest	territory	nest	territory	nest	territory	nest	territory	nest	territory	nest	territory
Baseline	0	0	0	0	0	0	0	0	0	0	0	0
12 h	5	6	0	1	2	4	1	1	0	3	2	4
24 h	6	6	1	0	1	2	0	0	0	0	0	2
36 h	3	5	0	0	1	0	0	0	0	0	0	0
48 h	3	5	0	0	2	0	0	1	0	0	0	0
60 h	1	2	1	0	0	0	0	0	0	0	0	0
72 h	0	3	1	0	0	0	0	1	0	0	0	0

### Results of baseline measurements

Baseline recordings of HR, IBI, SDNN, ACT and BT showed similar levels from day to day in each individual. Baseline curves of body weight, food and water consumption exhibited a few outliers, mainly in water bottle weighing. Analysis of cage sketches showed that all animals had built a proper nest and had established clear structuring of their habitat during the 3-day stabilization period. In all cases, the condition of the nest and the overall territorial structure was maintained during the baseline phase.

### Deviations of telemetrically measured parameters

**Figure 1:**
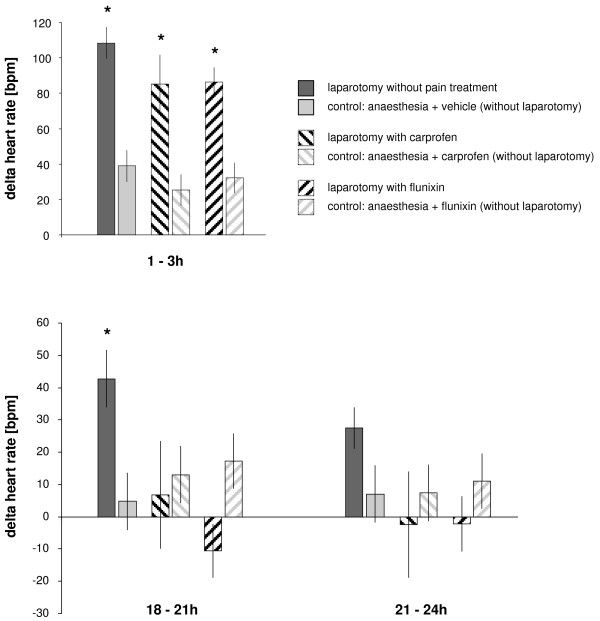
**Changes in heart rate immediately following laparotomy**. Changes (delta) in heart rate (HR) immediately after laparotomy and at the end of the first day after laparotomy are presented. Bars represent 3-hour means (± SEM). Asterisks indicate statistical significance (paired Student's *t *test with Bonferroni correction) at *P *≤ 0.016.

Figure 1 illustrates the mean changes in HR measured in the 3-hour period immediately after the animals were returned to their home cage after anaesthesia/laparotomy, and in two 3-hour periods at the end of the first day, i.e., 18–21 h and 21–24 h; during all three time intervals, the highest values were recorded in mice that had undergone laparotomy without subsequent pain treatment (*P *= 0.001 at 1–3 h; *P *= 0.008 at 18–21 h; *P *= 0.060 at 21–24 h). During the first 3 hours after recovery from anesthesia, the groups treated with carprofen (*P *= 0.002 at 1–3 h) or flunixin (*P *= 0.003 at 1–3 h) showed increased values, but by the end of the first day HR was normalized in the treated groups, whereas values remained elevated in the group in which analgesics were withheld after laparotomy.

**Figure 2:**
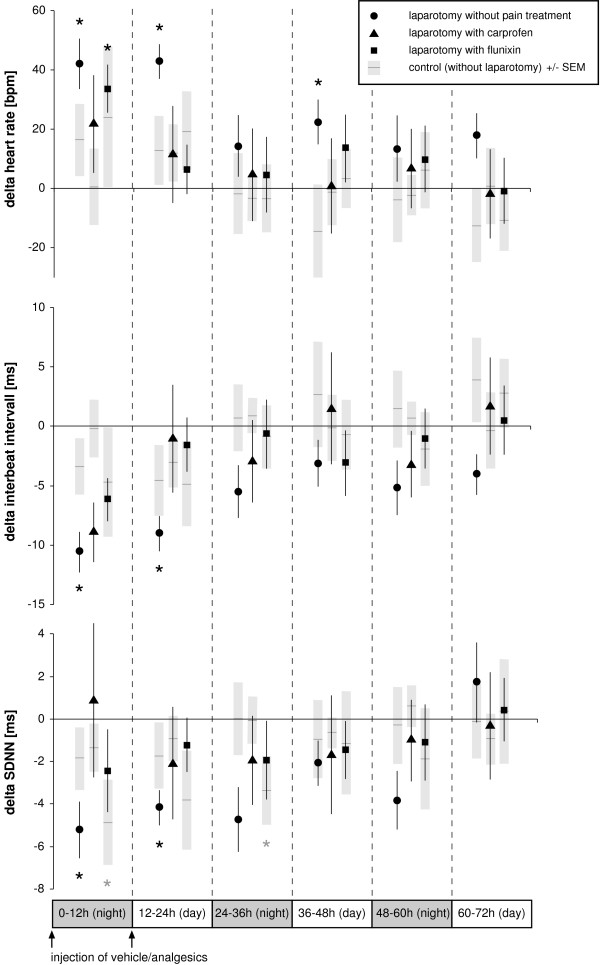
**Time course analysis of heart rate and heart rate variability following laparotomy**. Changes (delta) in heart rate [HR, beats per minute (bpm)], interbeat interval [IBI, milliseconds (ms)], and standard deviation of interbeat interval [SDNN, milliseconds (ms)] relative to baseline (i.e., normal values taken the day before the experiment) are plotted over time. Symbols indicate 12-hour means (bars indicate ± SEM). Corresponding control experiments in which animals received anesthesia and injections only are depicted as black horizontal lines with grey columns representing ± SEM. Asterisks indicate statistical significance (*n *= 8, paired Student's *t *test with Bonferroni correction) at *P *≤ 0.008. Note increased heart rate values with decreased heart rate variability parameters [IBI, SDNN] during the first light phase (12–24 h) in operated animals without pain treatment.

Plotting the results as 12-hour means over the 3 days following the experimental procedure (Figure 2) revealed that HR was significantly increased in the laparotomy group without pain treatment during the first 24 hours (*P *= 0.001 at 0–12 h; *P *= 0.000 at 12–24 h) and again in the second light phase (*P *= 0.001 at 36–48 h). HR was also significantly increased in the first dark phase in the laparotomy group treated with flunixin (*P *= 0.003 at 0–12 h).

A significant decrease in HRV was observed 24 hours after laparotomy without pain treatment, measured both as IBI (*P *= 0.004 at 0–12 h, *P *= 0.002 at 12–24 h) and SDNN (*P *= 0.006 at 0–12 h, *P *= 0.008 at 12–24 h). SDNN also decreased during the dark phase in the control group that received anaesthesia and flunixin treatment without laparotomy (*P *= 0.007 at 0–12 h, *P *= 0.002 at 24–36 h).

**Figure 3:**
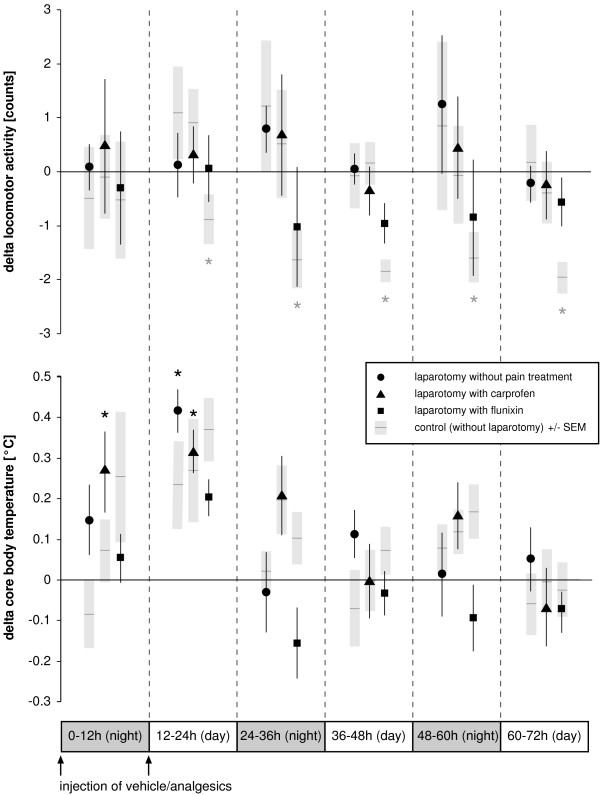
**Time course of locomotor activity and core body temperature following laparotomy**. Changes (delta) in locomotor activity and core body temperature relative to baseline (i.e., normal values taken the day before the experiment) are plotted against time. Symbols represent 12-hour means (bars indicate ± SEM). Corresponding control experiments in which animals received anesthesia and injections only are depicted as black horizontal lines with grey columns representing ± SEM. Asterisks indicate statistical significance (*n *= 8, paired Student's *t *test with Bonferroni correction) at *P *≤ 0.008.

ACT and BT after laparotomy and control experiments are reported in Figure 3. No alteration of ACT was found following laparotomy. Only the control group that underwent anesthesia and flunixin treatment showed a decline from 12 h onwards, with *P *values ranging from 0.002 to 0.006. BT increased for 24 h after laparotomy with carprofen treatment (*P *= 0.002 at 0–12 h and 12–24 h) and from 12–24 h in the group that was untreated after laparotomy (*P *= 0.001).

### Changes of body weight, food and water intake

**Figure 4:**
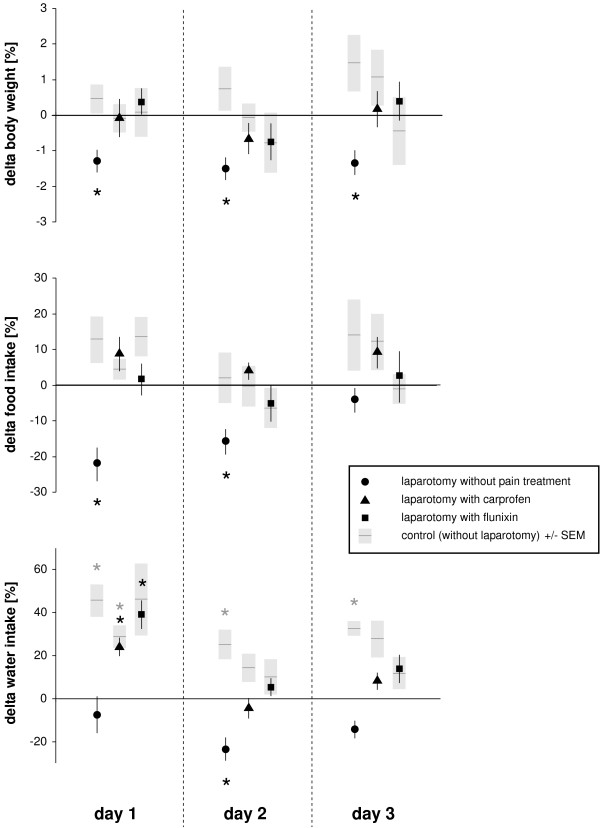
**Daily body weight, food and water intake**. The percentage deviation (delta) from baseline of body weight, and food and water intake is presented. Symbols represent the mean values from 8 animals, with bars indicating ± SEM. Corresponding control experiments in which animals received anesthesia and injections only are depicted as black horizontal lines with grey columns representing ± SEM. Asterisks indicate statistical significance (paired Student's *t *test with Bonferroni correction) at *P *≤ 0.016. Note the reduction in body weight for three days and the decrease in food consumption for two days after laparotomy without pain treatment.

Deviations in body weight, and food and water intake are illustrated in Figure 4. Body weights were significantly reduced for 3 days only in the group with laparotomy without pain treatment (*P *= 0.006 at day 1, *P *= 0.003 at day 2, *P *= 0.007 at day 3). In the same group, food consumption was reduced for two days (*P *= 0.004 at day 1, *P *= 0.007 at day 2). Water intake was decreased in this group, with significance at the second day (*P *= 0.005). Other groups showed increased water consumption after laparotomy and in control experiments, with significance values below 0.009.

### Summary of observations of body condition, behavior and territory

The criteria used to assess the general condition of the animals are listed in Table 1. A score of 1 could not be assigned to any of the experimental groups as determined from observations of the animal's body and general behaviour.

**Figure 5:**
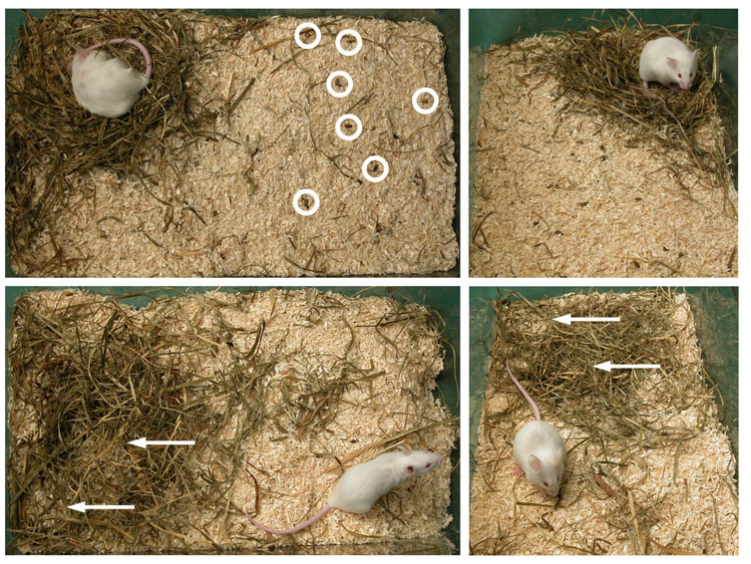
**Representative pictures of cage appearance**. The upper row indicates Score 0 with a well-built nest and clear structure of the cage area; feces is visible on the beddings surface (circles). The lower row illustrates Score 1 with an unstructured cage area and two nest-like resting places (arrows).

The results from assessment of the appearance of the cage are summarized in Table 2; representative pictures are presented in Figure 5. Unstructured territory, which persisted for 2 days, was detected mainly after laparotomy without pain treatment. Disintegration of the territory was mostly associated with signs of intense digging or the confused distribution of nesting materials in the habitat. In this group, a well-built nest was frequently missing, i.e. either no nest could be identified or several fragmentary nests at different locations were found during the first day.

## Discussion

In our model, telemetric recordings demonstrated significant alterations in heart action following laparotomy without pain treatment: HR was increased whereas HRV (IBI, SDNN) was decreased for 24 hours. When analgesics were applied, these parameters remained stable at normal, baseline levels, suggesting that the observed alterations in HR and HRV were associated with pain. This result was corroborated by measurements of body weight and food intake, both of which were significantly reduced if analgesics were omitted after laparotomy. Cage appearance supported an effect of the pain relief regimens; damaged nests and unstructured territory were frequently found in the group in which analgesics were withheld after surgery. Thus, behavioral measures such as food intake and nest building, were in line with aberrant HR and HRV, thus supporting the hypotheses that these parameters can be used as evidence of post-laparotomy pain in our model.

In contrast, no signs of pain could be discriminated by observation and scoring of the animals' bodily condition and spontaneous or provoked moving behaviors. Accordingly, real-time locomotor activity levels were not influenced, confirming that physical exercise was not the cause of the altered HR and HRV measures.

From common knowledge, HR is expected to increase under pain, because autonomous heart actions are adapted by the nervous system to life circumstances such as fear, anxiety, and pain. An increased resting HR compared to healthy controls was described in patients suffering from chronic pain due to irritable bowel syndrome [14] or interstitial cystitis [15]. The short-term response of telemetrically measured HR has been described as a method for studying the analgesic properties of pharmaceutical compounds in an analgesiometric model of visceral pain in rats [16].

In mice, elevated telemetrically measured HR combined with depressed ACT and flattening of circadian rhythms was reported for 5–7 days following implantation of blood pressure transmitters [17]. The magnitude and duration of these alterations, together with the substantial tissue damage from surgery, clearly suggest that this intervention induced a more severe grade of pain than the laparotomy used in our model.

Severe or considerable pain is known to induce apathy, as demonstrated by decreased values of ACT [18,19]. We found no aberrations in ACT following laparotomy with or without pain relief. Therefore we conclude that, in our model, animals did not suffer severe pain but rather mild-to-moderate pain.

HR measures can be verified by time course analysis of various HRV parameters. One such parameter, the interbeat interval (IBI) detects the distance between R-peaks in the ECG curve, and therefore a decline in IBI confirms elevated HR values. The SDNN is the standard deviation of the IBI in each measuring segment, representing the variability in HR changes. Thus, the SDNN describes the balance between parasympathetic and sympathetic modulation of HR, the decreased values in animals without treatment following laparotomy indicating sympathetic activation [13,20]. Similar changes in HR and HRV parameters have been shown to be associated with the progress of laminitis, a disease that is known to cause considerable pain in horses [21].

Although HR and HRV parameters ought to be useful in assessing pain, they have rarely been taken into account because of their sensitivity to various unrelated influences. These additional factors range from experimental interventions, including anaesthesia or medication, to environmental conditions such as an unfamiliar situation (e.g., foreign surroundings, person or companion animal). Whereas pharmaceutical compounds can influence these parameters per se (e.g., by inducing bradycardia), external influences can additionally act on the animal by stimulation of bodily exercise (e.g., running), which naturally leads to an elevation of HR. Such hyperactivity can be due to various reasons, one of which, typical for rodents, is novelty. Increased activity is easily evoked by providing animals with a new cage, which is frequently practiced after surgery, but which stimulates mice to explore and occupy the new housing – expressed by physical efforts such as running/walking, digging, climbing and scent marking of the cage area [22]. The influence of a novel cage during the experiment might be an explanation as to why, in another study, evidence of increased HR levels was not provided after laparotomy in mice [23]. In contrast to previous studies, in the present experiments mice were returned to their one-week old cages for recovery following anaesthesia. The inability to identify any significant relationship between nest-damaging behaviour and increased physical exercise as evidenced by the unchanged values of telemetrically recorded ACT levels, suggests that this was not the cause of HR elevation. In addition, the control groups, which received anaesthesia and injections only, displayed minimal rumpling of their nest and cage area, thus supporting our interpretation that this behavior was an expression of discomfort and therefore was related to post-laparotomy pain.

BT was of less importance in our model of laparotomy, where temperatures predominantly increased only in the range of up to 0.5°C. These moderate changes were also demonstrated in the controls after 12 to 24 h. Although BT measurements were relatively uninformative, they did allow the exclusion of infection (serious inflammation and fever) as an additional factor.

The HR and HRV outcomes were confirmed by the measurements of body weight and food intake, which decreased significantly only if analgesics were omitted after laparotomy. The reduction in food consumption continued for up to 2 days, which is probably the cause for the extended loss of body weight up to 3 days. These symptoms are well-known in animals suffering pain [3,5,7,19,24-27], but they are suggested to be relatively insensitive, or not specific, to pain [6]. From the defined conditions applied in the present study, it is apparent that body weight and food intake are suggestive parameters of pain in our model, but they appear to be both less sensitive and less accurate, especially with regards to determining the duration of pain, compared to HR and HRV.

Water intake proved not to be useful in assessing the well-being of the animals. We found an increased demand for fluid after anesthesia, which seemed to be satisfied by increased water consumption. If animals are in pain, water consumption is suggested to be depressed [2,5,7,9], thus fluid homeostasis could be unbalanced if pain is not relieved. Although the curves of water intake supported this assumption, it should be stressed that these data are of limited value due to the fact that artifacts (arising from e.g., accidental loss of water from the bottle) were not taken into account.

Analysis of the descriptive sketches of the cages revealed aberrations only in the group without pain treatment following laparotomy. Many of these animals damaged their nests when they were returned to their home cage after vasectomy. Half of the nests were only properly re-built after more than 2 days and, in some cages, normal structuring of the cage into different areas (i.e., resting places, place for excretion) was not discernable. These aberrations peaked at 12–24 hours, and occurred only rarely in mice that received analgesics and in control mice. We concluded that this nest-destroying behavior was an expression of distress or pain.

The control mice in the experiments served to show whether the observed changes might indicate pharmacologic effects of anesthesia and analgesics that do not reflect pain relief. Control groups that underwent anesthesia and analgesic or vehicle injections without surgery exhibited no substantial effects on the parameters measured. Prolonged effects of anesthesia were excluded by using a short-acting inhalation anesthesia with sevoflurane. As analgesics, two NSAID were chosen, which, to our knowledge, have no relevant effects on the parameters measured here. Whereas no side effects were found with carprofen, flunixin depressed ACT in the control experiment in the long term (> 12 h) and SDNN in night phases. Since the effect of flunixin on the key measures, i.e. HR, IBI, food intake, body weight, nest building and territorial structure after surgery, in the control group was similar to that of carprofen, we concluded that analgesia was the prominent effect, but a sedative side effect could not be completely excluded in the case of flunixin. Concerning the increased HR under flunixin in the first night phase after surgery, we assume that the analgesic effect of flunixin could have been slightly delayed due to subcutaneous application.

In summary, moderate pain was evident for one day after laparotomy without pain relief in our model. Pain was effectively alleviated with carprofen or flunixin (5 mg/kg body weight, s.c., bid).

## Conclusion

Surgical intervention as laparotomy induced significant changes of heart rate and heart rate variability parameters (interbeat interval, SDNN) that last for 24 hours. In addition, a significant decrease of food intake during 2 days postoperatively resulted in a significant reduction of body weight for 3 days. Pain therapy with carprofen and flunixin inhibited the changings of heart rate and heart rate variability and maintained food consumption and therefrom body weight remained stable.

The results show that i.) the method of continuous telemetric monitoring of physiological parameters was able to identify signs of mild to moderate grade pain in mice, which could not be clearly detected otherwise in this species in real-time ii.) analgesic regimens acted successfully in relief of mild to moderate post-operative pain in mice.

In conclusion, heart rate and heart rate variability may be useful in assessing post-operative pain in laboratory mice – provided measurements are recorded continously under standardized conditions in an undisturbed environment. Telemetry was shown to be a useful tool for real-time monitoring of post-operative recovery in mice.

## Abbreviations

Heart rate (HR), electrocardiogram (ECG), core body temperature (BT), locomotor activity (ACT), time domain analysis of heart rate variability (HRV), interbeat interval (IBI), standard deviation of interbeat interval (SDNN).

## Authors' contributions

MA developed the project, conducted the experiments, analyzed the data and drafted the manuscript.

AR and HPK participated in the design of the study, performed statistical analysis and prepared the figures.

PC participated in performing experiments and acquisition and interpretation of data.

KB participated in coordination of the study and helped to draft the manuscript.

All authors read and approved the final manuscript.
